# Infectious Aortitis Due to *Salmonella* Species Masquerading as Lung Malignancy

**DOI:** 10.1016/j.jaccas.2023.101943

**Published:** 2023-07-10

**Authors:** John-Paul O’Shea, Darakhshan Ahmad, Dorothy Garner, Tasaduq N. Fazili

**Affiliations:** Virginia Tech Carilion School of Medicine, Roanoke, Virginia, USA

**Keywords:** infected aortic aneurysm, infectious aortitis, lung malignancy, mycotic aneurysm, *Salmonella* bacteremia

## Abstract

Infectious aortitis is a rare but devastating vascular infection with mortality exceeding 40%. Early diagnosis is crucial but often hampered by radiographic mimickers. We report a patient who was thought to have lung cancer but ultimately found to have an infected aortic aneurysm and bacteremia owing to *Salmonella* species. Owing to surgical contraindications, he was treated palliatively with an initial regimen of intravenous ampicillin/sulbactam followed by lifelong oral antibiotic suppression. He ultimately rejected his diagnosis, discontinued medications, and was lost to follow-up. (**Level of Difficulty: Intermediate.**)

## Central Illustration

**Figure:**
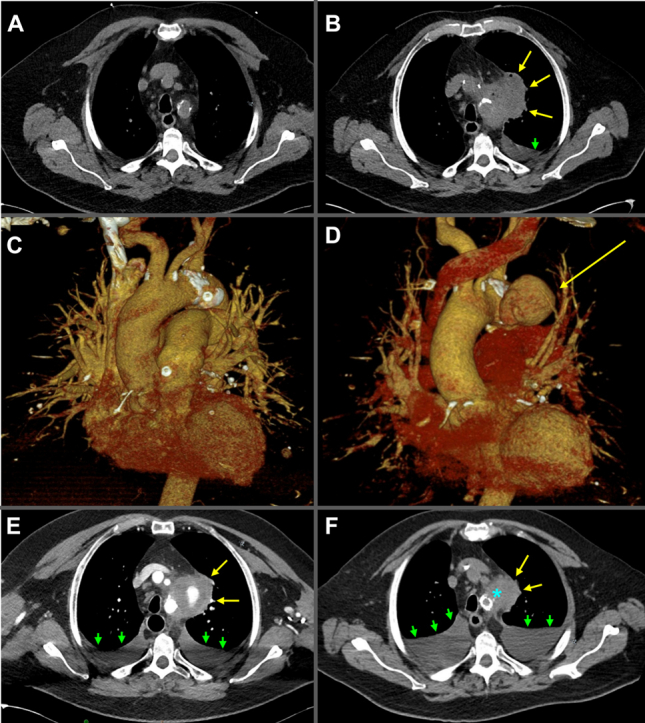

**Figure 1:**
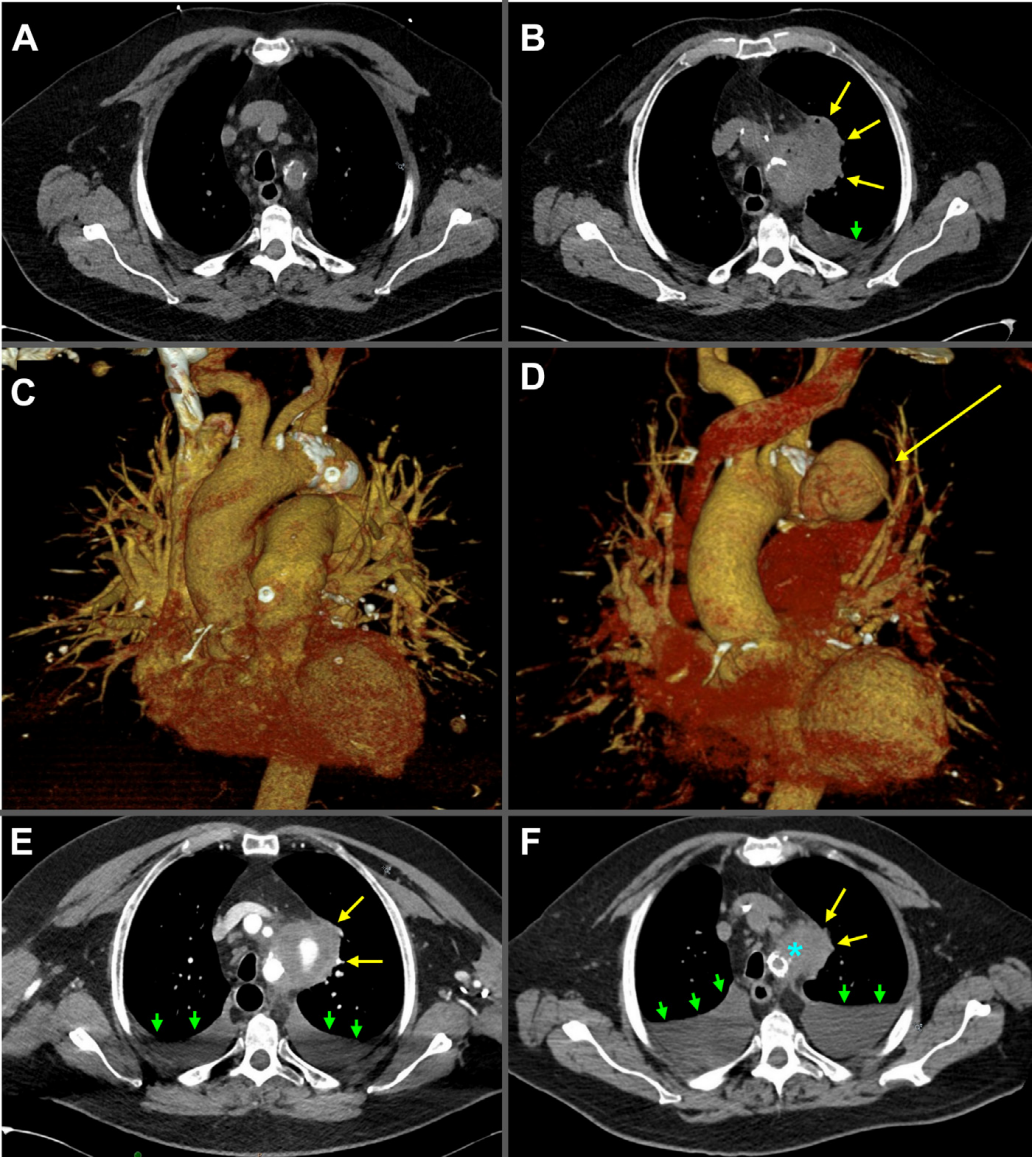
CT Cross-Sectional Imaging at Thoracic Vertebral Level 3/4 and CTA 3D Reconstructions, Demonstrating Radiographic Appearance and Rapid Evolution of the Aneurysm **(A, C)** Initial images with no aneurysm seen. **(C, D)** Two months, later showing the “lung mass” abutting the left mediastinum and a left pleural effusion. **(E)** At 3 months (before TEVAR), showing aneurysmal progression and bilateral pleural effusions. **(F)** At 6 months (after TEVAR) during an admission for heart failure exacerbation, showing aneurysm stability and progressive bilateral pleural effusions. **Yellow arrows** point to the saccular aneurysm of the proximal descending thoracic aorta. **Green arrowheads** point to pleural effusions. **Blue asterisk** indicates TEVAR. CT = computed tomography; CTA = computed tomography angiography; TEVAR = thoracic endovascular aortic repair.

A 75-year-old man with a 20-pack-year smoking history, heart failure (left ventricular ejection fraction of 30%-35%), coronary artery disease, diabetes mellitus, chronic kidney disease stage 3, recent COVID-19 pneumonia, and a strong family history of lung cancer, presented with syncope and abdominal pain associated with fevers, chills, rigors, and dyspnea. Although physical examination and basic laboratory tests were unremarkable, computed tomography (CT) of the chest demonstrated bilateral lung opacifications, without aortic dilatation, pericardial effusion, or pulmonary embolism. He was diagnosed with community acquired pneumonia and discharged on doxycycline (Figures 1A and 1C).

Two months later, he re-presented with a sore throat, dysphonia, dyspnea, and hemoptysis. Transthoracic echocardiography redemonstrated stable left ventricular systolic dysfunction but no obvious vegetations. CT angiography of the chest performed to investigate for pulmonary embolism showed a left mediastinal border opacification in addition to airway narrowing owing to right pyriform sinus and tonsillar pillar prominences (Figure 1B). He was discharged for outpatient workup of suspected lung cancer. Blood cultures eventually grew *Salmonella spp*, nontyphi, nonparatyphi, and he was readmitted to start ceftriaxone. CT angiography of the chest (Figures 1D and 1E) now revealed an approximately 5 × 6 cm, saccular aneurysm of the proximal descending thoracic aorta, with signs of impending rupture. With the concatenation of clinical history and findings—symptoms refractory to empiric antibiotics, rapid lesion growth, elevated inflammatory markers, and bacteremia with *Salmonella spp*—the differential diagnosis pivoted toward infectious aortitis rather than pulmonary abscess or malignancy. Owing to his comorbidities, he was considered a poor candidate for guideline-directed open surgical interventions (aortic arch resection with extra-anatomic reconstruction or in situ reconstruction).^1^ He alternatively underwent a palliative thoracic endovascular aortic repair (TEVAR) with zone 2 deployment. The distal aspect of the graft terminated proximal to the origin of the left vertebral artery with patency of the left common carotid and left subclavian arteries demonstrated on aortogram. He was discharged to continue 6 weeks of intravenous ampicillin/sulbactam followed by lifelong suppression with oral amoxicillin/clavulanic acid. Several months after his TEVAR, he was readmitted for an acute heart failure exacerbation and a surveillance CT of the chest demonstrated aneurysmal stability (Figure 1F). He followed-up with multiple providers who endorsed differing opinions regarding the etiology of his original decompensation, and he became convinced that he never had an aneurysm or bacteremia, and that his symptoms were due to long-COVID19. Despite robust counseling, he self-discontinued suppressive antibiotics and was lost to follow-up.

Infectious aortitis is a rare (approximately 3% of aortic aneurysms) but devastating vascular infection with *Salmonella* species (second to *Staphylococcus aureus*) as a common bacterial cause.^1^ Diarrheal episodes owing to *Salmonella* gastroenteritis may (remotely) precede typical presentations with fevers, dyspnea, and chest and abdominal pain. Endothelial injury owing to hypertension, diabetes mellitus, atherosclerosis, chronic kidney disease, and smoking place those who have had *Salmonella* exposure at risk (either through direct or indirect fecal–oral routes, eg, consumption of contaminated beef, poultry, milk, eggs; handling chickens or reptiles. After inoculation, *Salmonella* is phagocytosed by macrophages and together they become incorporated during atheroma formation, ultimately transforming into an infected aneurysm. Owing to the latency of the bacteria inside atheroma macrophages, blood and stool cultures often become negative, making microbiological diagnosis challenging.^2^ Therefore, imaging should be pursued when infectious aortitis is suspected but cultures remain negative. CT scans and magnetic resonance imaging have high pooled sensitivities (80%-90%) and can aid interventional planning.^3^ If these modalities are equivocal or the patient is at risk for contrast-induced kidney injury, using positron emission tomography with CT scans and tagged white blood cell scans can provide cumulative diagnostic clarity.^2^

High mortality rates with antibiotics alone (75%-100%) mandate the consideration of prompt source control with surgery.^1^ Although mortality with open surgery is approximately 21%, recurrence rates approximate 0%, compared with TEVAR (mortality approximately 7%, recurrence approximately 17%).^2^ Even when intervention is possible, owing to either challenges with source control or implantation of stents into an infected vasculature, prolonged antibiotic courses (>6 weeks) are indicated and, in the case of conservative approaches, lifelong.

Our patient vocalized that it was the mixed messages from various health providers regarding his diagnosis as the reason he became convinced that he did not have infectious aortitis and why he self-discontinued his suppressive antibiotics. The elevated mortality of infectious aortitis, together with the procedurally invasive and prolonged antibiotic courses required, make patient buy-in to their diagnosis and treatment strategy imperative if they are to have a chance at a successful outcome; interprovider communication toward presenting a uniform message is essential.

In conclusion, this case highlights that, in patients with *Salmonella* bacteremia, persistent symptoms despite empiric antibiotics and abnormal imaging, the differential diagnosis should include infectious aortitis. A multidisciplinary approach presenting a uniform message is essential for patient buy-in to their diagnosis and treatment strategy.

## Funding Support and Author Disclosures

The authors have reported that they have no relationships relevant to the contents of this paper to disclose.
